# Chemotherapy-Induced Degradation of Glycosylated Components of the Brain Extracellular Matrix Promotes Glioblastoma Relapse Development in an Animal Model

**DOI:** 10.3389/fonc.2021.713139

**Published:** 2021-07-19

**Authors:** Alexandra Y. Tsidulko, Oleg B. Shevelev, Anna S. Khotskina, Mariia A. Kolpakova, Anastasia V. Suhovskih, Galina M. Kazanskaya, Alexander M. Volkov, Svetlana V. Aidagulova, Evgenii L. Zavyalov, Elvira V. Grigorieva

**Affiliations:** ^1^ Institute of Molecular Biology and Biophysics, Federal Research Center of Fundamental and Translational Medicine, Novosibirsk, Russia; ^2^ Institute of Cytology and Genetics, Siberian Branch of the Russian Academy of Sciences, Novosibirsk, Russia; ^3^ V. Zelman Institute for Medicine and Psychology, Novosibirsk State University, Novosibirsk, Russia; ^4^ Meshalkin National Medical Research Center, Ministry of Healthcare of the Russian Federation, Novosibirsk, Russia; ^5^ Novosibirsk State Medical University, Ministry of Healthcare of the Russian Federation, Novosibirsk, Russia

**Keywords:** glioblastoma multiforme, chemotherapy, extracellular matrix, proteoglycan, glycosaminoglycan, chondroitin sulfate, temozolomide, dexamethasone

## Abstract

Adjuvant chemotherapy with temozolomide (TMZ) is an intrinsic part of glioblastoma multiforme (GBM) therapy targeted to eliminate residual GBM cells. Despite the intensive treatment, a GBM relapse develops in the majority of cases resulting in poor outcome of the disease. Here, we investigated off-target negative effects of the systemic chemotherapy on glycosylated components of the brain extracellular matrix (ECM) and their functional significance. Using an elaborated GBM relapse animal model, we demonstrated that healthy brain tissue resists GBM cell proliferation and invasion, thereby restricting tumor development. TMZ-induced [especially in combination with dexamethasone (DXM)] changes in composition and content of brain ECM proteoglycans (PGs) resulted in the accelerated adhesion, proliferation, and invasion of GBM cells into brain organotypic slices *ex vivo* and more active growth and invasion of experimental xenograft GBM tumors in SCID mouse brain *in vivo*. These changes occurred both at core proteins and polysaccharide chain levels, and degradation of chondroitin sulfate (CS) was identified as a key event responsible for the observed functional effects. Collectively, our findings demonstrate that chemotherapy-induced changes in glycosylated components of brain ECM can impact the fate of residual GBM cells and GBM relapse development. ECM-targeted supportive therapy might be a useful strategy to mitigate the negative off-target effects of the adjuvant GBM treatment and increase the relapse-free survival of GBM patients.

## Introduction

GBM is an aggressive malignant brain tumor with a very poor prognosis. The conventional treatment protocol includes surgery followed by concurrent radiotherapy (30 fractions*2 Gy) and chemotherapy with TMZ, with subsequent TMZ courses (1, 2). This adjuvant treatment targets the eliminiation of residual GBM cells and, in the most cases, accompanied by DXM as a basic anti-edema drug (3, 4). During the last decade, substantial efforts have been made to develop new anti-GBM drugs and different treatment strategies; however, they did not lead to significant improvement in survival rates of GBM patients yet (5, 6). Unfortunately, despite the temporal improvement in the disease course, a GBM relapse can develop at 6 to 8 months after surgery resulting in a lethal outcome. At recurrence, there is no standard of care, and there is a clear need for better therapeutic options (7, 8).

The known molecular mechanisms of adverse TMZ effects on the residual GBM cells are presented in detail in the comprehensive reviews (9, 10) and include largely induced resistance of GBM cells to the drug(s) due to DNA repair (11, 12) and contribution of cancer stem-like cells (13–16). For the last years, the tumor microenvironment (TME) also appeared on this scene as an important regulator of phenotypic GBM cells heterogeneity and tumorigenic potential rather than multipotency of cancer stem-cell cells (17).

The role of the microenvironment in brain cancer has been extensively studied during last decades, but that is related, initially, to the role of cellular components of the brain tissue, like the astrocytes (18, 19). ECM components remain less investigated, although their importance to GBM development is not in doubt (20). Brain tissue ECM represents a unique structure, mainly composed of proteoglycans (PGs) and glycosaminoglycans (GAGs), which involvement in normal physiology and carcinogenesis is extensively studied for different cancers (21, 22). The important role of PGs in brain carcinogenesis is comprehensively discussed in the recent reviews (23–25), with special attention to chondroitin sulfate proteoglycans (CSPGs) (26, 27) and CSPG4 (28), heparan sulfate proteoglycans (HSPGs) (29), and polysaccharide molecules of heparan sulfate (HS) (30–32) and keratan sulfates (KS) (33). As for GAGs, one of the most abundant GAGs in brain ECM is hyaluronan (HA), which has a functional role in the GBM development and is comprehensively described in the recent review by Pibuel et al. (34).

During GBM chemotherapy, TMZ affects PGs expression in both the residual cancer cells and surrounding normal brain tissue. More data are available for TMZ effects on PGs in GBM tumor/cells because of the possibility of using experimental cancer cell culture systems *in vitro* and *in vivo* as well as clinical data. Important results are obtained from the experiments with TMZ-resistant GBM cells, which allow us to identify potential resistance-related PGs: glypican-1–silenced U-251 cells were much more susceptible to TMZ than intact U-251 MG cells (35); high expression of decorin and lumican in GBM and neuroblastoma stem cells are associated with TMZ resistance (36); CSPG4 is related in molecular mechanisms of TMZ resistance of GBM cells (37); experimental degradation of CS by chondroitinase ABC sensitizes glioblastoma cells to TMZ (38). The observed changes in the PGs expression in TMZ-resistant GBM cells seem to be induced by the systemic long-term TMZ treatment during the selection of the resistant cell clones. Overall, although the use of TMZ in the experimental models does not reflect the clinical situation of GBM development completely (39, 40), the experiments allow for the revealing of important molecular mechanisms of GBM development and identifying PGs as markers for GBM cells transformation.

Effects of systemic long-term TMZ on the extracellular components of normal brain tissue (PGs, GAGs) remain much less investigated due to restricted set of the research methods, which include mainly GBM animal models *in vivo* (41–44) and brain organotypic culture *ex vivo* (45–47). In our previous study, we demonstrated for the first time the ability of TMZ and DXM to affect PG/GAG expression and composition in normal rat brain tissue in the experimental system *in vivo*. TMZ treatment affects mainly GAG chains of the intact PG molecules, while DXM increases overall transcriptional activity and expression patterns of the PGs (changes in syndecan-1 (+4-fold), glypican-1 (+3-fold), brevican (+7-fold), CSPG4/NG2 (+2-fold), decorin (−2-fold), and lumican (+3-fold) expression) in brain zone-dependent manner (48). Combination of TMZ with DXM results in the most profound deterioration in PGs composition and content in the brain tissue both at core protein and glycosaminoglycan levels. In this study, we aimed to investigate the functional role of the TMZ-induced changes in the expression of PGs and content of their polysaccharide GAG chains in GBM relapse development using a novel GBM relapse model.

## Materials and Methods

### Animals

For *in vivo* studies, male SCID mice (n=64) aged 10 weeks and weighing 23 to 30 g were used. Animals were housed in groups of two to five mice in individually ventilated polycarbonate cages OptiMice (Animal Care Systems, USA) in special clean rooms with HEPA13-filtered incoming air, with free access to food and water, 12/12-h light/dark cycle, air temperature of 22 ± 2°C, and relative humidity of 45 ± 10%. All *in vivo* experiments were conducted at SPF Animal Facility at the Institute of Cytology and Genetics SB RAS (Novosibirsk, Russia). For *ex vivo* organotypic hippocampal slice culture, Wistar rat pups aged 8 to 9 days were used. All procedures were conducted in accordance with the European Communities Council Directive 2010/63/EU and were approved by the Animal Care and Use Committees of the Institute of Cytology and Genetics SB RAS and FRC FTM. All efforts were made to minimize animal suffering and to reduce the number of animals used.

### Cells

The human glioblastoma U87 cell line was obtained from the Karolinska Institute (Stockholm, Sweden). The U87-RFP cell line was purchased from Creative Biogene (Shirley, NY, USA). Cells were maintained in IMDM medium supplemented with 2 mM l-glutamine, 100 units/ml penicillin, 100 μg/ml streptomycin, and 10% fetal bovine serum at 37°C in a humidified 5% CO_2_ incubator. For analysis, cells were harvested using trypsin/EDTA.

### Primary Mixed Glial Culture

Primary mixed glia was obtained using gentleMACS™ Octo Dissociator with Heaters and Adult Brain Dissociation Kit (Miltenyi Biotec, Germany) according to the manufacturer’s protocol. Briefly, two 10-week-old C57BL/6J mice were sacrificed by cervical dislocation; brains were removed and washed in cold PBS. Brains were cut into 0.5-cm slices and transferred to C Tubes (Miltenyi Biotec, Germany), containing enzyme mix 1. Enzyme mix 2 was added, and the brains were dissociated using the gentleMACS program 37C_ABDK_01. After the termination of the program, the suspension was filtered through a 70-μm strainer and centrifuged at 300*g* for 10 min. Debris was removed using centrifugation with Debris Removal Solution at 3000*g* for 10 min. Cells were washed with cold PBS, incubated with 1× Red Blood Cell Removal Solution for 10 min at 4°C and centrifuged at 300*g* for 10 min. The remaining cells were resuspended in IMDM (Gibco, USA) medium supplemented with 2 mM l-glutamine, 100 units/ml penicillin, 100 μg/ml streptomycin, and 10% fetal bovine serum. Cells were seeded onto poly-d-lysine-covered flasks and incubated at 37°C in a humidified 5% CO_2_ incubator. The medium was changed completely after 24 h, and ½ of the medium was changed every 3 days. At day 11, cells reached 85% to 90% of confluency and were used for further experiments.

### Cell Viability and Proliferation Assay

Cell viability and proliferation were detected by Hoechst 33342/PI staining. Primary mixed glial cells were seeded on 96-well plates at 10^4^ cells per well and allowed to attach and grow for 24 h. After 24 h medium was changed to the medium containing TMZ (250 µM), DXM (1 µM), TMZ+DXM (250 and 1 µM, respectively) or DMSO (0.25%) as control, ½ medium was replaced with fresh medium containing corresponding drugs every 3 days. Cells viability and proliferation were analyzed at 24, 48, 72, 144, 168, and 192 h of incubation with the drugs. Cells were stained with Hoechst 33342 (Sigma-Aldrich) for 30 min at 37°C and PI (Sigma-Aldrich, USA) for 10 min at 37°C. An IN Cell Analyzer 2200 (GE Healthcare, UK) was used to perform automatic imaging of six fields per well under 200× magnification, in bright field and fluorescence channels. The images produced were used to analyze live and dead cells using the IN Cell Investigator software (GE Healthcare, UK).

### RT-PCR Analysis

Total RNA was extracted from the brain samples and organotypic hippocampal slices using the TRIzol Reagent (Thermo Fisher Scientific, USA) and RNeasy Plus Mini kit (Qiagen, USA) according to the manufacturer’s instructions. cDNA was synthesized from 1 μg of total RNA using a first strand cDNA Synthesis kit (Fermentas, USA). Quantitative real-time RT-PCR was performed using the CFX96 Real-Time PCR Detection System (Bio-Rad, USA) and the PCR iTaq Universal SYBR Green Supermix (Bio-Rad, USA) under the following conditions: 95°C for 2 min, followed by 40 cycles at 95 °C for 10 s and 60°C for 30 s. The total reaction volume was 25 µl. The relative amount of mRNA was normalized against Gapdh mRNA, and the fold change for each mRNA was calculated by the 2^−ΔCt^ method. Primer sequences for rat and mouse genes are presented in **Table 1**.

**Table 1:** Sequences of primers used in PCR analysis.

Description	GeneBank	Gene	Organism	Sequence
Syndecan-1	NM_013026.2	Sdc1	*Rattus norvegicus*	F 5’-GAACCCACCAGCAGGGATAC-3’
				R 5’-CACACTTGGAGGCTGATGGT-3’
	NM_011519.2	Sdc1	*Mus musculus*	F 5’-GGTCTGGGCAGCATGAGAC-3’
				R 5’-GGAGGAACATTTACAGCCACA-3’
Glypican-1	NM_030828.1	Gpc1	*Rattus norvegicus*	F 5’-GCCAGATCTACGGGGCTAAG-3’
				R 5’-AGACGCAGCTCAGCATACAG-3’
	NM_016696.5	Gpc1	*Mus musculus*	F 5’-CTTTAGCCTGAGCGATGTGC-3’
				R 5’-GGCCAAATTCTCCTCCATCT-3’
Perlecan	XM_017593851.1	Hspg2	*Rattus norvegicus*	F 5’-TGATGACGAGGACTTGCTGG-3’
				R 5’-ACACCACACTGACAACCTGG-3’
	NM_008305.3	Hspg2	*Mus musculus*	F 5’-CCGTGCTATGGACTTCAACG-3’
				R 5’-TGAGCTGTGGAGGGTGTATG-3’
Versican	NM_001170558.1	Vcan	*Rattus norvegicus*	F 5’-ATGTGGATCATCTGGACGGC-3’
				R 5’-GTTTCGATGGTGGTTGCCTC-3’
	NM_001081249.1	Vcan	*Mus musculus*	F 5’-GGAGGTCTACTTGGGGTGAG-3’
				R 5’-GGGTGATGAAGTTTCTGCGAG-3’
Brevican	NM_012916.2	Bcan	*Rattus norvegicus*	F 5’-AGGGGACCTCACAAGTTCTTC-3’
				R 5’-ATTTGACTCGGGGAAAGCCC-3’
	NM_012916.2	Bcan	*Mus musculus*	F 5’-GTGGAGTGGCTGTGGCTC-3’
				R 5’-AACATAGGCAGCGGAAACC-3’
CSPG4/NG2	NM_031022.1	Cspg4	*Rattus norvegicus*	F 5’-ATCTGGGAGGGGGCTATTGT-3’
				R 5’-GTACGCCATCAGAGAGGTCG-3’
	NM_139001.2	Cspg4	*Mus musculus*	F 5’-TCTTACCTTGGCCCTGTTGG-3’
				R 5’-ACTCTGGTCAGAGCTGAGGG-3’
CD44	NM_009851.2	Cd44	*Mus musculus*	F 5’-CAAGTTTTGGTGGCACACAG-3’
				R 5’-AGCGGCAGGTTACATTCAAA-3’
Decorin	NM_024129.1	Dcn	*Rattus norvegicus*	F 5’-AATGCCATCTCCGAGTGGTG-3’
				R 5’-TTGTCGTGGAGTCGAAGCTC-3’
	NM_007833.6	Dcn	*Mus musculus*	F 5’-CCCCTGATATCTATGTGCCC-3’
				R 5’-GTTGTGTCGGGTGGAAAATC-3’
Biglycan	NM_017087.1	Bgn	*Rattus norvegicus*	F 5’-GAACAGTGGCTTTGAACCCG-3’
				R 5’-CCTCCAACTCGATAGCCTGG-3’
	NM_007542.5	Bgn	*Mus musculus*	F 5’-GCCTGACAACCTAGTCCACC-3’
				R 5’-CAGCAAGGTGAGTAGCCACA-3’
Lumican	NM_031050.1	Lum	*Rattus norvegicus*	F 5’-AATTTGACCGAGTCCGTGGG-3’
				R 5’-GCCTTTCAGAGAAGCCGAGA-3’
Neurocan	NM_007789.3	Ncan	*Mus musculus*	F 5’-CCAGCGACATGGGAGTAGAT-3’
				R 5’-GGGACACTGGGTGAGATCAA-3’
Gapdh	NM_017008.4	Gapdh	*Rattus norvegicus*	F 5’-ATGGCCTTCCGTGTTCCTAC-3’
				R 5’-TCCAGGGTTTCTTACTCCTTGG-3’
	NM_008084.3	Gapdh	*Mus musculus*	F 5’-CGTCCCGTAGACAAAATGGT-3’
				R 5’-TTGATGGCAACAATCTCCAC-3’

### Immunostaining

For immunohistochemistry, 3-μm sections of formalin-fixed, paraffin-embedded tissue samples were used. Deparaffinization and antigen retrieval were performed in PT Module with Dewax and HIER Buffer L (Thermo Scientific, USA). Tissue sections were stained using Lab Vision™ Autostainer 720-2D according to the UltraVisionQuanto HRP DAB Protocol (Thermo Scientific, USA). Briefly, sections were incubated with UltraVision Hydrogen Peroxide Block buffer for 10 min RT and then UltraVision Protein Block solution for 5 min RT before being incubated with primary mouse monoclonal antibody to CS-AC (1:100, CS-56, C8035, Sigma-Aldrich, USA) for 1 h RT. The signal was visualized by incubations with Primary Antibody Amplifier Quanto (10 min, RT), HRP Polymer Quanto (10 min, RT), and DAB Quanto solutions (5 min, RT). All washing steps were performed with Tris-buffered saline and Tween-20 buffer (Thermo Scientific, USA). Staining patterns were counterstained with hematoxylin and photographed by light microscopy with magnification ×400 (AxioScope.A1 with AxioCamMRc5 (Carl Zeiss, Germany).

### Dot-Blots for Chondroitin Sulfate Content

Brain tissue samples were lysed with RIPA-buffer (Thermo Scientific, USA), containing “Complete” Protease Inhibitor Cocktail (Roche, USA), sonicated, and centrifuged for 15 min at 14,000*g*. The protein concentration was quantified using Pierce™ BCA Protein Assay Kit (Thermo Scientific, USA). 1 μg of total proteins were dot-blotted onto PVDF membranes in a volume of 1 μl. The membranes were blocked with 5% non-fat milk for 1 h and incubated with mouse anti-CS primary antibody (1:500, CS-56, C8035, Sigma-Aldrich, USA) overnight at 4°C followed by secondary peroxidase-conjugated antibodies goat anti-Mouse IgG (Abcam, UK) for 1 h at RT. GAGs were detected with an Optiblot ECL Detection Kit (Abcam, UK) according to the manufacturer’s instructions. Blots were imaged using ChemiDoc (BioRad, USA) and analyzed semi-quantitatively using ImageJ 1.52 software.

### Organotypic Hippocampal Slice Culture *Ex Vivo*

Organotypic hippocampal slice cultures (OHSCs) were prepared according to the previously described protocol (49). Briefly, neonatal Wistar rat pups (postnatal days 8–9) were decapitated, and the brains were rapidly removed under aseptic conditions and placed into ice-cold Hank’s balanced solution with 0.9% glucose. The hippocampi were removed and cut rapidly into 400-μm transversal slices with a manual McIlwain tissue chopper (Stoelting Co., USA). The slices were transferred to Millicell culture inserts (Millipore, PICM0RG50) placed into six-well plate containing 1.2 ml of culture medium, consisting of 30% Hank’s balanced solution, 60% IMDM and 10% fetal bovine serum. The organotypic hippocampal slices were cultivated in a 90% humidified atmosphere with 5% CO_2_ at 37°C. The medium was changed the next day. At the day 4 of the experiment, ½ of the medium was replaced with neurobasal medium supplemented with B27 (Gibco, USA), at day 7, medium was completely replaced with Neurobasal+B27 medium and changed twice a week.

TMZ and/or DXM were added to the culture medium to final concentrations 250 and 1 µM, respectively, at day 7 of the experiment, the treatment conditions were taken from our previous work (48). The organotypic hippocampal slices were incubated with drugs for 24 h, washed with fresh medium and used for co-culture with glioma cells or collected into RNA*Later* solution for RT-PCR analysis.

### GAG Content Manipulation in Rat Brain Organotypic Slices

To degrade endogenous GAGs in the organotypic slices, enzymes chondroitinase AC (EC 4.2.2.5, Sigma-Aldrich, USA), and chondroitinase B (EC 4.2.2.19, Sigma-Aldrich, USA) were used. Chondroitinase AC (0.5 U/ml) or chondroitinase B (30 U/ml) were resuspended in the reaction buffer (150 mM NaCl, 25 mM K_2_PO_4_, pH 6.5) and applied to brain organotypic slices on the day 8 of the experiment in a volume of 10 μl/slice. The slices were incubated for 1 h at 37°C and 5% CO_2_, washed with fresh culture medium and used to co-culture with glioblastoma U87-RFP cells.

To increase the amount of GAGs in the organotypic culture, endogenous CS-A/C (Sigma-Aldrich, USA) or CS-B (Sigma-Aldrich, USA) were added to the culture medium on the 8th day of the experiment to a final concentration 0.5 mg/ml. The content of exogenous GAGs in the medium was maintained during subsequent cultivation with GBM U87-RFP cells.

Validation of the used reagents’ specificity is provided on the technical specification inserts.

### Co-Culture of Organotypic Hippocampal Slices With U87 GBM Cells

U87-RFP cells were harvested using trypsin/EDTA, pelleted by centrifugation, and resuspended in Neurobasal medium.

To analyze the adhesion of tumor cells to the surface of organotypic slices, 12500 cells in 10 μl were applied onto each slice, incubated for 2 h at 37°С and 5% CO_2_, washed in PBS, and fixed in 10% neutral buffered formalin at 4°C for 16 h. To assess the proliferation of tumor cells and their invasion into organotypic slices, 2,500 cells in 5 μl were applied onto each slice, incubated for 7 days at 37°С and 5% CO_2_, then washed with PBS and fixed in 10% neutral buffered formalin at 4°C for 16 h. After fixation, organotypic slices-U87-RFP co-cultures were washed three times in PBS, transferred onto microscope slides, and covered with a coverslip using SlowFade Gold medium with DAPI (Thermo Scientific, USA). These co-cultures were visualized using an LSM 710 laser confocal microscope (Carl Zeiss, Germany). Images were acquired in z-stack and tile-scan modes to visualize the signal over the entire volume of the slice. The acquisition, processing, and analysis of images were performed using the ZEN Black 2012 software (Carl Zeiss, Germany). Tumor cell adhesion was determined as the percentage of the slice surface area occupied by tumor cells (summarized signal from 20 µm deep slice volume); the proliferation of tumor cells was determined as a percentage of the area occupied by tumor cells in the maximum intensity projection mode (summarized signal from the whole slice volume); the invasion of tumor cells was determined as a percentage of the area occupied by tumor cells at a depth of 25 µm from the slice surface.

### Drug Administration to Healthy SCID Mice *In Vivo*

In total, 64 male SCID mice were randomly divided into four experimental groups. TMZ-group (n = 16) received TMZ (MSD, Finland) intragastric as a water suspension in a dose of 30 mg/kg; DXM-group (n=16) received intraperitoneal injection of 1 mg/kg DXM (KRKA, Slovenia), TMZ-DXM-group (n=16) received both TMZ and DXM; control group received water intragastric in the same volume as the TMZ-group. The drugs were administered according to the scheme: three cycles of 5 consecutive days of administration with a 9-day break between cycles (a total of 15 drug injections). The animals were weighed once a week. On the 39th day of the experiment, six animals from each group were sacrificed by cervical dislocation; the brains were removed, one hemisphere was divided into cerebral cortex and subcortex and collected in RNA*Later* solution (Invitrogen, USA) for RT-PCR analysis, the second hemisphere was incubated in 10% neutral buffered formalin for 24 h at room temperature and used to prepare paraffin blocks.

The remaining animals received an orthotopic injection of human GBM U87 cells.

### Orthotopic Experimental Tumors Development in the Pre-Treated SCID Mice Brain

The experimental human GBM tumors were induced in the brain of the pre-treated SCID mice by a stereotactic inoculation of U87 cells into the subcortical brain structures (50). Briefly, mice were placed into a chamber with 1.5% isoflurane and airflow of 450 to 500 ml/min for 3 min, and then transferred onto a 37°C heated operating table and placed under an anesthesia mask with 1.5% isoflurane. A 3- to 4-mm incision on the head skin was made in the caudal-cranial direction in the bregma area, and 5 μl of U87 cells suspension in serum-free DMEM/F12 medium (5 × 10^5^ cells per animal) was injected into the subcortical brain structures with a Hamilton syringe through a hole in the skull. The experimental tumor growth was monitored by magnetic resonance imaging (MRI) every 5 days starting at day 10 after tumor cells inoculation using a BioSpec 117/16 USR horizontal tomograph (Bruker, Germany) at 11.7 T using a TurboRARE (Rapid Imaging with Refocused Echoes) T2 scanning sequence (TR=2500 ms, TE_eff_=24 ms, NA=5, Rare factor = 8, matrix 256×256 dots, field of view 2.0 cm × 2.0 cm). All manipulations were performed on anesthetized animals (1.5% isoflurane in a gas mixture with oxygen and a flow rate of 350–450 ml/min). Tumor size was calculated using the Paravision 5.1 (Bruker) and ImageJ software and expressed in μl. Mice were sacrificed upon 20% weight loss by decapitation using guillotine according AVMA Guidelines for the Euthanasia of Animals (American Veterinary Medical Association, 2013); no hunching, rough coat, ataxia, head tilt, and paralysis were detected. Brains were removed, one hemisphere was divided into the cerebral cortex, subcortex, and tumor and collected into RNA*Later* for RT-PCR analysis, the other hemisphere was fixed in 10% neutral buffered formalin and used to prepare paraffin blocks.

### Statistical Analysis

ANOVA analysis with Fisher’s least significant difference (LSD) *post hoc* test was performed to determine statistical significance between the studied groups. A value of p<0.05 was considered to indicate a statistically significant difference. Data are expressed as means ± SD. Survival analysis was performed by the Kaplan-Meier method. Pearson correlation coefficient was determined to analyze the correlation between PGs expression and tumor volume. All statistical analyses were performed using OriginPro 8.5 software.

## Results

Because our previous data indicated that TMZ and DXM change PGs structure and composition in normal rat brain tissue (48), we performed a number of functional tests to investigate whether the structural ECM changes affect a fate of GBM cells in pre-treated brain microenvironment and contribute to experimental tumor development mimicking a GBM relapse. Two complementary approaches were combined in a GMB relapse mouse model—xenograft tumor growth in immunocompromised mice *in vivo* and co-culture of GBM cells with brain slice cultures *ex vivo* that closely mimic tumor cell invasion into the brain *in vivo* (46).

### TMZ and DXM Facilitates Adhesion, Proliferation, and Invasion of GBM Cells Into Rat Brain Organotypic Slices *Ex Vivo*

**Figure 1:**
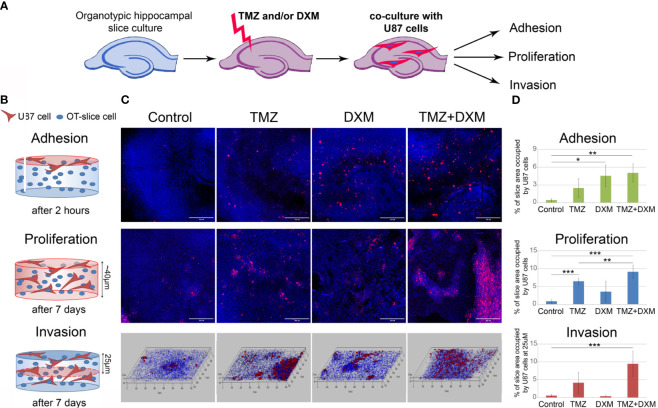
Effects of TMZ and/or DXM-induced changes in brain organotypic slices on adhesion, proliferation and invasion of GBM cells *ex vivo*. **(A)** Scheme of the experiment. **(B)** Methodology for detection of the studied parameters. **(C)** Confocal microscopy of U87-RFP cells seeded on the control and TMZ/DXM-treated organotypic brain slices. Cells nuclei are stained with DAPI. Magnification, ×200, scale bar 500 µm. **(D)** Quantitative analysis of the U87-RFP cells on the control and treated rat brain tissues. ANOVA and post-hoc Fisher’s LSD test, *p < 0.05, **p < 0.01, ***p < 0.001. TMZ, temozolomide; DXM, dexamethasone.

First, we performed the experiment on co-culture of organotypic brain slices with U87-RFP cells (**Figure 1A**). The hippocampal slices were treated with TMZ and/or DXM, and the drugs were removed from the culture medium before addition of U87 cells. TMZ/DXM-induced changes in PGs expression were verified by real-time RT-PCR analysis (**Supplementary Figure 1**). Adhesion, proliferation, and invasion of the fluorescent-labeled cells on the pre-treated brain tissue were assessed as shown (**Figure 1B**), representative pictures are presented (**Figure 1C**), and staining signal was quantified using ImageJ 1.52 software (51) (**Figure 1D**). It was shown that TMZ and DXM possess different effects on brain ECM structure. Although DXM-induced changes facilitated adhesion of GBM cells to the pre-treated hippocampus slices (4- to 5-fold, p<0.05), TMZ-induced changes favored the proliferation of the cells (7-fold, p<0.001) (**Figures 1C, D**). The most drastic deterioration of brain ECM occurred upon combined TMZ/DXM treatment that resulted in significant activation of U87 adhesion (4- to 5-fold p< 0.01), proliferation (9- to 10-fold, p< 0.001), and invasion (9- to 10-fold, p< 0.001) to the pre-treated brain organotypic culture. These data demonstrate that healthy brain tissue is capable of suppressing adhesion and proliferation of cancer cells, but the systemic use of TMZ (especially in combination with DXM) attenuates this ability and contributes to the transformation of brain microenvironment into a pro-invasive niche. Together, these results reveal the overall toxic effects of systemic use of TMZ (especially in combination with DXM) to brain tissue structure in terms of PGs/GAGs pattern and content, and their impaired ability to resist cancer cells adhesion and proliferation in experimental system *ex vivo*.

### Pre-Treatment of SCID Mice With TMZ and DXM Increases Growth and Invasive Potential of Xenograft U87 Tumors *In Vivo*

**Figure 2:**
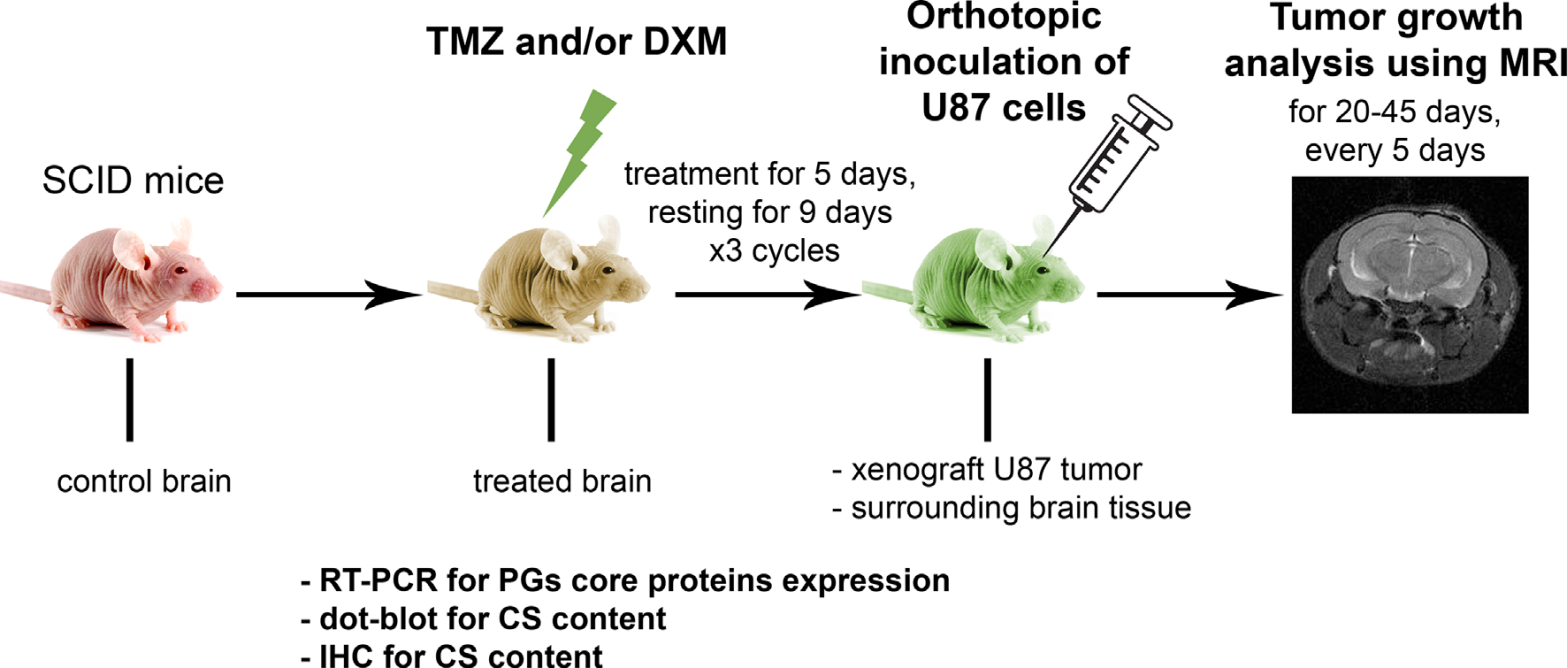
Experimental GBM relapse animal model. Scheme of the experiment to study the effects of pre-treatment of SCID mice with chemotherapeutic drugs on the growth of xenograft U87 tumors. PG, proteoglycan; CS, chondroitin sulfate; TMZ, temozolomide; DXM, dexamethasone.

**Figure 3:**
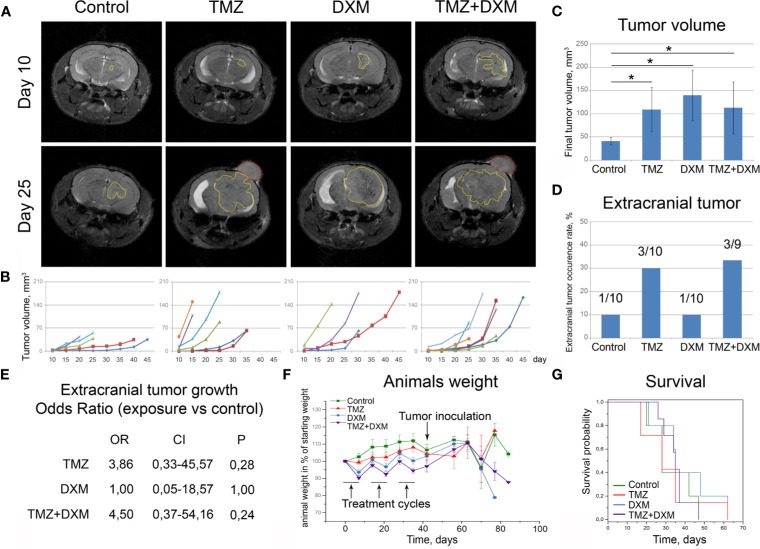
Functional effects of TMZ and/or DXM-induced changes in brain tissue on the development of experimental U87 xenograft tumors. **(A)** MRT-images of the representative U87 xenografts. Intracranial tumors marked with a yellow outline, extracranial—red outline. **(B)** Growth curves for the xenografts in control and treated brains. **(C)** Final volume of the xenografts. ANOVA and post-hoc Fisher’s LSD test, *p < 0.05. **(D)** Frequency of the extracranial tumors in all experimental groups. **(E)** Odds ratio of extracranial tumors development (OriginPro 8.5). **(F)** Weight of the animals during the experiment. **(G)** Survival of the animals (Kaplan-Meier curve). TMZ, temozolomide; DXM, dexamethasone.

To investigate the effects of TMZ chemotherapy *in vivo*, we elaborated a mouse model of GBM relapse, where healthy animals were first treated with TMZ and/or DXM, and then the glioma U87 cells were inoculated into the brain pre-exposed to chemotherapeutic drug(s) (**Figure 2**). This model mimics a clinical situation where post-surgery residual GBM cells have to survive and proliferate in the microenvironment compromised by a long-term TMZ pressure. Indeed, we observed a significant (2.5- to 3-fold) increase in the growth rate and final volume of xenograft U87 tumors in the animals that have undergone TMZ or DXM treatments compared with control animals (**Figures 3A–C**). Other things being equal, normal brain tissue possessed a restraining effect on tumor growth *in vivo*, being similar to the results obtained in the *ex vivo* model system.

Interestingly, the xenograft tumors grown in the animals that received TMZ or combined TMZ-DXM treatment demonstrated not only the bigger tumor size but also more invasive phenotype as well, seen on MRI images, unlike those with DXM-treatment (**Figure 3A**). They developed extracranial tumors at significantly higher rates (**Figure 3D**), supporting further the conclusion about an accelerated invasion of GBM cells into the TMZ-compromised brain tissue. The calculated odds ratio showed a 3.8- to 4.5-fold higher probability of extracranial tumor formation in the brain of TMZ-treated animals (**Figure 3E**). At the same time, the final body weight of the mice from different experimental groups did not differ (although some fluctuations following the TMZ/DXM treatments occurred), and overall survival rates were similar (**Figures 3F, G**).

These *in vivo* results match to the data obtained in brain organotypic slices *ex vivo* and demonstrate different effects of TMZ and DXM on healthy brain tissue, where TMZ pre-treatment (especially in combination with DXM) facilitates adhesion, proliferation, and invasion of GBM cells into the surrounding brain tissue and active growth of the experimental tumors.

### TMZ and/or DXM Affect PGs Expression in Normal Mouse Brain Tissue

**Figure 4:**
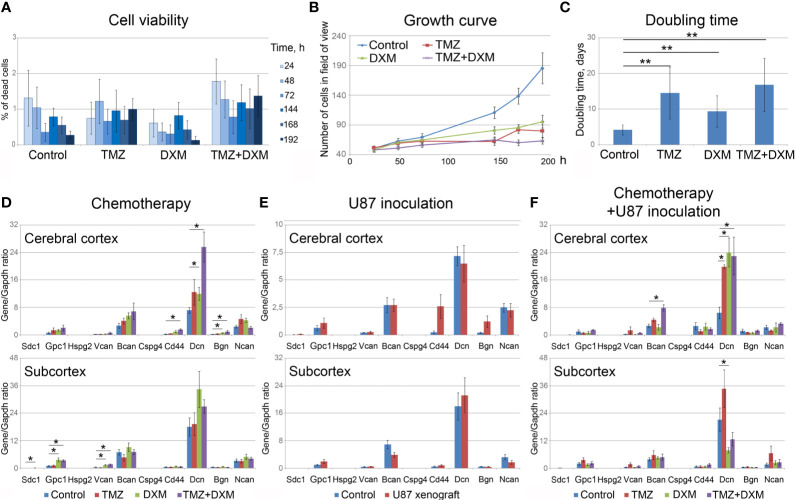
Effects of TMZ and/or DXM on normal glial cells and proteoglycans expression in brain tissue. **(A)** Viability of primary glial cells during the treatments (as a percentage of dead cells in the culture). **(B)** Growth curves of the control and TMZ/DXM-treated cells. **(C)** Doubling time for the control and treated cells. **(D, E)** PG core proteins mRNA levels in cortex and subcortex before and after treatments with TMZ and/or DXM **(D)** or U87 cells inoculation **(E)** or combination of TMZ/DXM treatments and inoculation of U87 cells **(F)**. Real-time RT–PCR analysis, intensity of the amplified DNA fragments normalized to that of *Gapdh*. Bars represent the mean ± SD from triplicate experiments (OriginPro 8.5). ANOVA + Fisher’s LSD test, *p < 0.05, **p < 0.01. TMZ, temozolomide; DXM, dexamethasone.

To analyze the effect of TMZ or DXM on cellular components of normal mouse brain tissue, the viability of primary mixed glial cells from mouse brain and their proliferation rate upon TMZ/DXM treatment were analyzed using InCell Analyzer 2000 System (**Figure 4**). None of the treatments had a significant effect on the amount of dead cells in the culture (**Figure 4A**), although their proliferative activity was inhibited (**Figures 4B, C**). TMZ/DXM treatment significantly affected the expression of some PG core proteins in the brain of healthy SCID mice in brain zone-specific manner (**Figure 4D**), whereas U87 xenograft tumor growth did not significantly change PGs’ expression in the brain of the SCID mice (**Figure 4E**). The most significant changes were observed after combined treatment with TMZ/DXM (**Figure 4F**). DXM increased mRNA levels of biglycan (2.7-fold, p<0.05) in the cortex, and glypican-1 (3.5-fold, p<0.01), syndecan-1 (4.3-fold, p<0.05), and versican (3.1-fold, p<0.001) in the subcortex. TMZ alone did not significantly affect the transcriptional activity of PG-coding genes in the brain tissue, but in combination with DXM resulted in a completely different pattern of PG expression: up-regulation of biglycan (4.3-fold, p<0.05), CD44 (6.3-fold, p<0.01), and decorin (3.6-fold, p<0.01) in cortex and increased expression of glypican-1 (3.2-fold, p<0.01) and versican (3.7-fold, p<0.001) in subcortex. The obtained results demonstrate that TMZ/DXM selectively affect the expression of some PGs at the mRNA levels, resulting in the specific PGs expression patterns and deteriorated brain ECM composition.

### TMZ and/or DXM Decrease CS Content in Normal Mouse Brain Tissue

**Figure 5:**
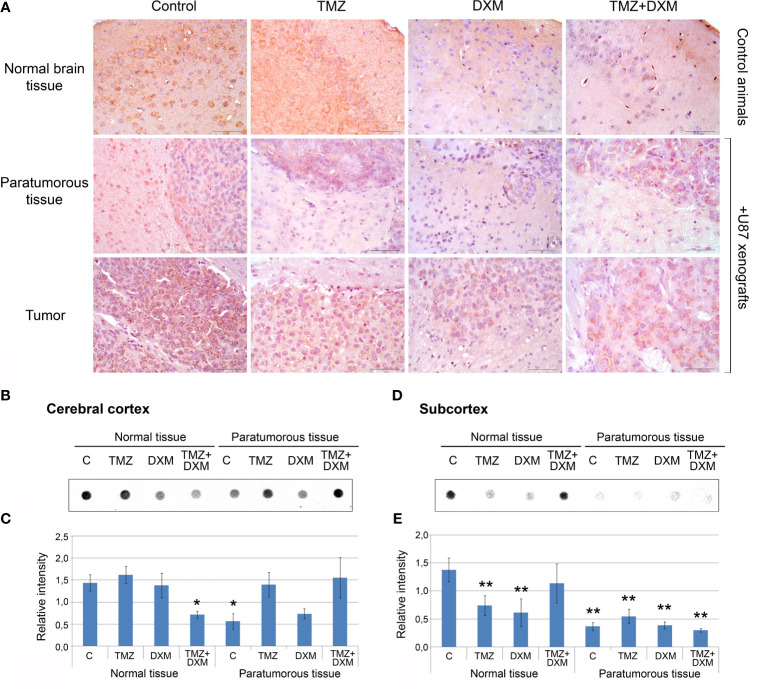
Chondroitin sulfate content in SCID mice brain tissues before and after treatments with TMZ and/or DXM. **(A)** Immunohistochemical analysis of CS content before and after TMZ/DXM treatments in normal SCID mouse brain tissue, paratumorous tissue and U87 xenografts. Magnification *400. Scale bars 50 µm. **(B–E)** Dot-blot analysis of the total CS content using anti-CS antibody in cortex and subcortex structures. **(B, D)** Original representative dot blots. **(C, E)** Semi-quantitative analysis of the dot-blots (ImageJ 1.52 software). Bars represent the mean ± SD from triplicate experiments (OriginPro 8.5). ANOVA + Fisher’s LSD test, *p < 0.05, **p < 0.01. Control, non-treated mouse brain tissue; TMZ, temozolomide; DXM, dexamethasone.

Because the functional properties of complex PG molecules to a large extent depend on their GAG chains, and decorin and brevican are the most expressed PGs in mouse brain tissue responding to TMZ/DXM treatment, we next analyzed the content of CS-AC in the TMZ/DXM-treated brain tissues. According immunohistochemical analysis (IHC) of brain tissue samples (include cerebral cortex and subcortex structures), CS is abundant in healthy SCID mice brain tissue, but combined TMZ/DXM treatment significantly decreased total CS-AC content (−2.3-fold, p<0.05) (**Figure 5A**, upper line). Inoculation of U87 GBM cells into the mouse brain resulted in the decrease of CS-AC content in paratumorous tissue to approximately the same level as that in TMZ/DXM-treated brain tissue, and this effect was even more pronounced when U87 xenografts were developed in TMZ/DXM pre-treated animals (**Figure 5A**, middle line). The U87 xenograft tumors themselves retained a high level of CS-AC content, mainly in the GBM cells (**Figure 5A**, lower line).

Quantitative analysis of the changes in CS-AC content was performed using dot-blot analysis with CS-AC–specific antibody (clone CS-56) for cerebral cortex and subcortex tissues separately (**Figures 5B–E**, respectively). Cerebral cortex was relatively resistive to the manipulations and showed significant decrease of CS content only after combined TMZ/DXM treatment (−2-fold, p<0.05) or U87 inoculation (−2-fold, p<0.05) (**Figures 5B, C**). Subcortex was more sensitive to any influence (TMZ, DXM, U87 inoculation with/without drug pre-treatments) demonstrating −2- −4-, 5-fold decrease in CS-AC content (p<0.01) (**Figures 5D, E**).

Together, these data support a hypothesis that both GBM cells and TMZ treatment contribute to the reorganization of brain ECM towards a pro-tumorigenic and pro-invasive microenvironmental niche, being more susceptible to GBM relapse development, through the attenuation of CS-AC content in brain ECM.

The obtained results for the first time indicate PGs/GAGs as potential targets for chemotherapeutic drug(s) in brain tissue, possibly involved in its transformation to a pro-tumorigenic niche.

### Xenograft U87 Tumors Growth Is Associated With PGs Expression in Mouse Brain Tissue

To investigate whether these TMZ/DXM-induced changes in PGs expression are associated with the increased proliferation and invasion of U87 cells, we used two complementary approaches.

**Figure 6:**
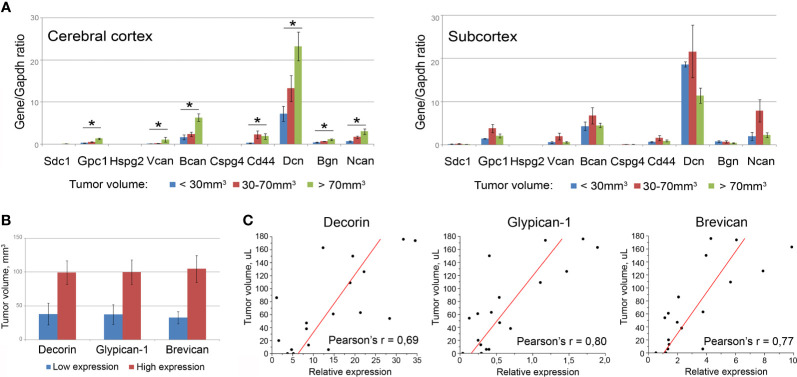
Association of the PGs expression levels in surrounding tumor normal tissue with the total volume of U87 xenografts. **(A)** PG expression levels in brain tissue of animals grouped according to the xenograft tumors size (small <30 µl; medium 30-70 µl; big >70 µl). Bars represent the mean ± SD from triplicate experiments (OriginPro 8.5). ANOVA + Fisher’s LSD test, *p < 0.05. **(B)** Tumor volumes in animals with relatively high or low expression of selected PGs (decorin, brevican, glypican-1). **(C)** Pearson’s linear correlation of the xenograft volumes with the expression levels of the PGs in surrounding normal brain tissue (OriginPro 8.5).

First, all the mice bearing U87 xenografts were allocated to three groups with relatively small (<30 µl), medium (30–70 µl), or big (>70 µl) tumors, and PG expression in the brain tissue surrounding these xenografts was analyzed separately in each of the cohorts (**Figure 6A**). Indeed, the normal tissue surrounding bigger-sized U87 tumors possessed significantly higher mRNA levels for multiple PGs, such as decorin (+3.2-fold, p<0.01), biglycan (+2.7-fold, p<0.05), glypican-1 (+4.7-fold, p<0.01), syndecan-1 (+3.4-fold, p<0.05), brevican (+3.8-fold, p<0.01), NG2/CSPG4 (+4.9-fold, p<0.01), neurocan (+4.7-fold, p<0.05), compared with the normal tissue surrounding small tumors.

Then, the whole experimental cohort was analyzed according to the high or low expression level of each individual PG and xenograft size in these animals. The transcriptional activity of three of the studied PGs (decorin, glypican-1, brevican) demonstrated a clear tendency to be correlated with the size of the experimental xenograft tumors (**Figure 6B**). To verify this observation, correlation analysis was performed for these best candidate PGs and confirmed a high correlation between the U87 xenograft volumes and the expression levels of the PGs surrounding the normal mouse brain tissue: decorin (Pearson’s correlation coefficient r=0.69), brevican (Pearson’s r=0.77), and glypican-1 (Pearson’s r=0.80) (**Figure 6C**).

These data suggest that TMZ-induced changes in the PGs expression in brain tissue contribute to the increased proliferative and invasive capacity of GBM cells in TMZ-compromised microenvironment.

### Degradation of Chondroitin Sulfate but Not Dermatan Sulfate Results in the Accelerated Adhesion and Invasion of GBM Cells Into Brain Tissue

**Figure 7:**
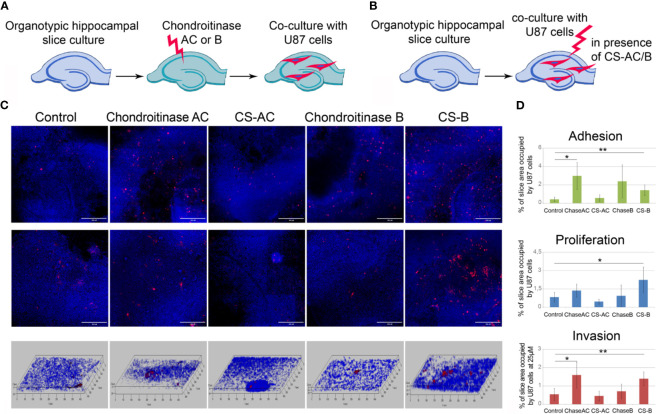
Effects of experimental modulation of CS content on adhesion, proliferation and invasion of GBM cells *ex vivo*. **(A, B)** Schemes of the experiment. **(C)** Confocal microscopy of U87-RFP cells seeded on the control organotypic brain slices and slices treated with chondroitinase AC or exogenous chondroitin sulfate AC (CSAC), chondroitinase B or chondroitin sulfate B (CSB). Cells nuclei are stained with DAPI. Scale bar 500 µm. **(D)** Quantitative analysis of the U87-RFP cells on the control and treated rat brain tissues (ImageJ 1.52 software). ANOVA and post-hoc Fisher’s LSD test, *p < 0.05, **p < 0.01. TMZ, temozolomide; DXM, dexamethasone.

To study a functional role of CS in GBM cells fate in the pre-treated microenvironment further, we used two approaches, consisting of the removal of different CS sub-types (CS-A/C or CS-B, called also dermatan sulfate, DS) by chondroitinase AC or chondroitinase B treatments (**Figure 7A**) and addition of exogenous CS-AC or CS-B (**Figure 7B**). The enzymes (chondroitinase AC and chondroitinase B) have been reported to be specific for the native CS polysaccharide molecules. They degrade specifically CS sub-types A/C and B, respectively, and may be used for identification of these CS sub-types in tissues and cells.

It was shown that enzymatic degradation of CS-AC in the organotypic brain slices before their co-culture with U87 cells increased adhesion and invasion but not proliferation of U87 cells on these pre-treated slices, whereas addition of exogenous CS-AC to the intact co-cultures of brain slices with U87 cells did not affect any of the studied functional characteristics of the GBM cells (adhesion, proliferation, migration) (**Figures 7C, D**). Pre-treatment of the brain slices with chondroitinase B did not affect the interaction of U87 cells with the brain tissue, although the exogenous CS-B significantly increased adhesion, proliferation, and migration of U87 cells (**Figures 7C, D**). The results suggest that CS-AC is a vital component, and an optimal balance of CS-AC and CS-B is required for the ability of healthy brain tissue to resist glioblastoma cells proliferation and invasion.

Taken together, the obtained results for the first time demonstrate that the TMZ-induced changes of PGs expression and CS-AC/CS-B content directly contribute to the accelerated proliferation and invasion of GBM cells into the compromised brain tissue and xenograft tumor growth in the used GBM relapse model.

## Discussion

It is known that after adjuvant chemoradiotherapy, GBM commonly recurs around the tumor removal site. It was suggested by Hide and Komohara that the microenvironment at the tumor border (“border niche”) provides therapeutic resistance to residual GBM cells that allows them to survive and recur at the tumor border, and its understanding is critical to prevent GBM relapse (52). Investigation of GBM TME, especially its extracellular components, demands specific approach related to the preservation of brain tissue ECM.

Currently, multiple glioma models were developed to investigate molecular mechanisms of GBM development, drug resistance of GBM cells, and role of cancer-initiating cells in these processes (41, 42). However, the number of models for studying GBM microenvironment is much smaller (44), and this is also the case for the number of models that investigate molecular mechanisms of GBM relapse development (43). In common, these parameters were investigated separately like studying cellular components (astrocytes) in the recurrent tumors grown after the resection of the primary glioma xenografts (43) or investigation of glioma cells invasion into normal brain slice cultures (45–47). In this study, we suggested a novel GBM relapse model combining mouse experiments *in vivo* and organotypic brain culture *ex vivo*, where normal brain tissue was pre-modified by TMZ and/or DXM before the inoculation of GBM cells to mimic GBM relapse development after extensive adjuvant chemotherapy (in contrast to conventional glioma models in which drug treatments are usually performed after the inoculation of cancer cells). Compared with other approaches, a specificity of this model is in the joining GBM relapse study and investigation of the role of glycosylated extracellular components of brain tissue in organotypic brain culture *ex vivo* pre-treated with TMZ/DXM. This approach allows obtaining original data on the involvement of brain TME in the GBM relapse development and can be useful for further research in this field.

Our results for the first time demonstrate that TMZ-induced deterioration of PGs expression and degradation of polysaccharide molecules CA-AC (but not CS-B) in normal brain tissue may represent a molecular mechanism for survival of the GBM cells and their active proliferation and invasion in the surrounding pre-treated brain tissue resulting in the relapse tumor development. These results add a piece of knowledge to the scarce published data on this issue and stay in line with the findings that CSPGs-rich brain ECM is associated with a noninvasive phenotype of GBM tumors, whereas low CSPGs content is more common for infiltrating tumors (26, 27). The effects are realized through activation of tumor-associated microglia and tumor encapsulation (26) and regulation of the dynamics of the CSPG binding with its receptor LAR (27) and are attributed to the complex CSPG proteoglycan molecules. At first glance, contradictory results were shown by Logun et al., showing that blockade of sulfated polysaccharide CS chains by the sulfated GAG antagonist surfen reduces adhesion and invasion of GBM cells in 3D composite highly sulfated CSA/E matrices. However, this effect was much weaker for low- or non-sulfated matrices and suggested that the functional effects of CSPGs to GBM cells invasion depend on CS sub-types and their sulfation (53). Together with different functional effects of CS-AC or CS-B (dermatan sulfate) degradation on the proliferation and invasion GBM cells shown in this study, these data perfectly correspond to the known differential functional properties of CS/DS molecules with different sulfation level (54, 55) and underline a necessity of careful consideration of CS/DS sub-types in further research on glioma cells behavior and GBM relapse development.

Overall, our findings indicate that long-term TMZ treatment affects the polysaccharide components of brain tissue ECM, transforming that into the pro-carcinogenic niche and creating a favorable microenvironment for GBM relapse development. A balance between the targeted effects and negative side effects of the systemic TMZ treatment toward brain ECM might have a principal mean for GBM relapse development and needs further investigation.

## Data Availability Statement

The original contributions presented in the study are included in the article/**Supplementary Material**. Further inquiries can be directed to the corresponding author.

## Ethics Statement

The animal study was reviewed and approved by Animal Care and Use Committees of the Institute of Cytology and Genetics SB RAS and FRC FTM.

## Author Contributions

Conceptualization was performed by AT and EG. Investigation was performed by AT, OS, AK, MK, AS, GK, and AV. Data curation and analysis were performed by AT, SA, EZ, and EG. The writing and editing were performed by AT, SA, and EG. All authors contributed to the article and approved the submitted version.

## Funding

This study was supported by the Russian Science Foundation (grant 16-15-10243) in part of SCID mice *in vivo* experiments and Russian Foundation for Basic Research (grant 18-29-01036) in part of molecular effects of therapy on CS pathway. AT was supported by scholarship of Russian Federation President for young scientists (SP-5435.2018.4). AS was supported by a scholarship of Russian Federation President for young scientists (SP-1816.2019.4).

## Conflict of Interest

The authors declare that the research was conducted in the absence of any commercial or financial relationships that could be construed as a potential conflict of interest.
