# Supplementary material for: Chemotherapy-Induced Degradation of Glycosylated Components of the Brain Extracellular Matrix Promotes Glioblastoma Relapse Development in an Animal Model
Source: Front Oncol. 2021 Jul 19;11:713139. doi: 10.3389/fonc.2021.713139 (PMC8327169; doi:10.3389/fonc.2021.713139)

Supplementary Material

## Supplementary Figure 1

Fold changes of proteoglycans expression levels in rat organotypic hippocampus culture treated with TMZ and/or DXM compared to that in control brain tissue. Real-time RT–PCR analysis, intensity of the amplified DNA fragments normalised to that of *Gapdh*. *- p<0.05.


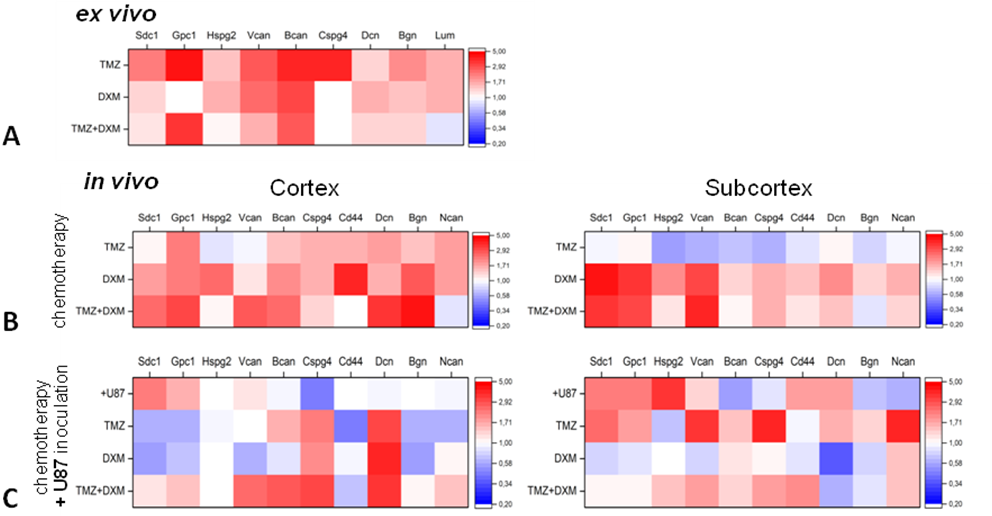

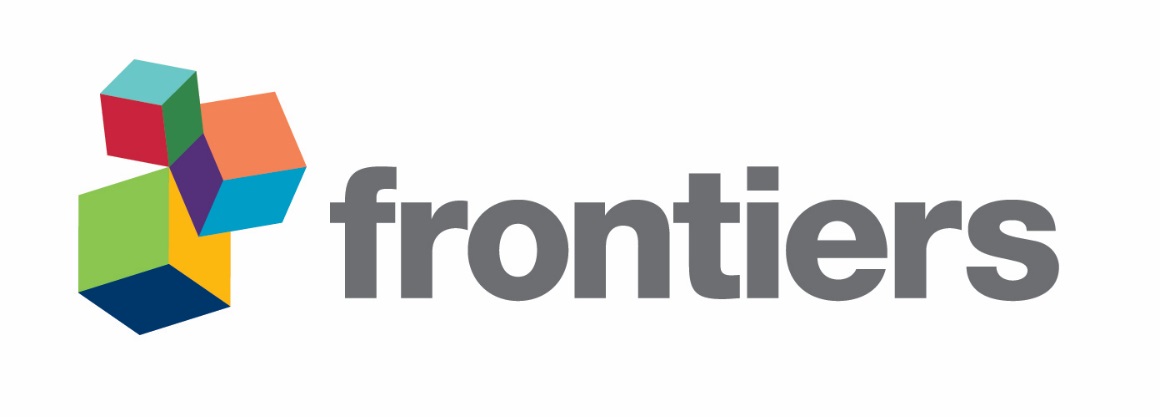

Supplement: Supplementary file 1 [file DataSheet_1.docx]
